# PBDE Flame Retardants and Thyroid Hormones: Chevrier et al. Respond

**DOI:** 10.1289/ehp.1002782R

**Published:** 2010-12

**Authors:** Jonathan Chevrier, Kim G. Harley, Asa Bradman, Brenda Eskenazi, Myriam Gharbi

**Affiliations:** Center for Children’s Environmental Health Research, School of Public Health, University of California, Berkeley, Berkeley, California, E-mail: chevrier@berkeley.edu; Pôle d’Épidémiologie et Santé Publique, Institut Pasteur, Paris, France

Goodman et al. raise concerns regarding our study (Chevrier et al. 2010) in which we reported associations between serum levels of polybrominated diphenyl ethers (PBDEs) and lower thyroid-stimulating hormone (TSH) in pregnant women participating in the Center for the Health Assessment of Mothers and Children of Salinas (CHAMACOS) cohort study. The points they raise are unlikely to have affected the validity of our conclusion.

Goodman et al. correctly point out that, as for most health outcomes, thyroid hormone levels may be affected by a number of factors. However, these factors must be related to PBDE serum levels to confound the associations that we reported. Although it would be of interest to evaluate associations between exposure to PBDEs and the adverse health effects cited by Goodman et al. (e.g., psychiatric disorders, depression, neurotransmitter and hormonal alterations), we are aware of no study that investigated these relationships. Furthermore, should PBDEs cause these health effects, they would be on the causal pathway between exposure and disease, and as such, should not be adjusted for. Consequently, it is unlikely that the factors identified by Goodman et al. substantially confounded our results. On the other hand, we considered a large number of demographic and environmental factors that may be related to both PBDE serum levels and thyroid function, and we observed little confounding (Chevrier et al. 2010).

Goodman et al. also suggest that our results may be due to exposure and outcome misclassification based on *a*) the reference range that we used to determine subclinical hyperthyroidism; *b*) our use of different cutoffs for women in the second and third trimesters of pregnancy; and *c*) the fact that we measured PBDEs at one time point during pregnancy. As explained below, we believe that substantial misclassification is unlikely; even if misclassification did occur, it would most likely be nondifferential with regard to exposure, which would be expected to bias associations toward the null rather than create spurious relationships.

Goodman et al. argue that in our study (Chevrier et al. 2010) we used inappropriate reference ranges to determine subclinical hyperthyroidism. Reference ranges for thyroid hormone are method, instrument, and gestational-age specific. The trimester-specific reference ranges that we used were developed by Quest Diagnostics on a Bayer ADVIA Centaur system (Siemens Healthcare Diagnostics, Deerfield, IL), the instrument used for our analyses; thus, they were appropriate. Reference ranges cited by Goodman et al., however, were based on studies that used different methods and instruments and/or that were conducted earlier in pregnancy (e.g., ≤ 21 weeks of gestation) (Haddow et al. 2004; Price et al. 2001; Stricker et al. 2007; as reviewed by Krassas et al. 2010).

Goodman et al. criticize our use of different reference ranges for women whose TSH was measured in the second and third trimesters of pregnancy. However, the National Academy of Clinical Biochemistry (Baloch et al. 2003), as well as authors cited by Goodman et al. (Krassas et al. 2010), recommend using trimester-specific reference ranges because thyroid hormone levels change during the course of pregnancy. Furthermore, we found inverse associations between PBDEs and TSH expressed continuously (Chevrier et al. 2010), demonstrating that the relationships are not due to the cutoff points used to dichotomize TSH.

Goodman et al. also believe that we should have taken multiple blood samples to determine exposure to PBDEs. The PBDE congeners that we measured, however, are highly persistent in humans (estimated half-lives of 2–12 years). In addition, PBDE serum concentrations measured at 27 weeks of gestation and at delivery were strongly correlated (*r* = 0.82–0.99; *p* < 0.001) among CHAMACOS women, suggesting that a single measurement is sufficient to determine exposure.

Goodman et al. state that the fact that congeners are intercorrelated leaves the impression that several congeners may be related to TSH. Correlation between the congeners was expected because they are components of the same commercial mixture (pentaBDE). Although identifying the specific congener(s) that may be related to thyroid hormone disruption is of scientific interest, it is of little relevance in terms of public health; relationships between any one of the measured PBDE congeners and thyroid hormone levels is of concern.

Finally, Pop et al. (1999) reported that small variations in maternal thyroid hormone during pregnancy were related with altered child neurodevelopment. In our study (Chevrier et al. 2010), we reported a 37.7% reduction in TSH over the full range of total PBDEs and a 3.9-fold increase in the odds of subclinical hyperthyroidism among women in the fourth quartile of BDE-100 relative to those in the first quartile. These associations are neither “very small” nor are they within the reference range, as argued by Goodman et al.

PBDE serum concentrations in the CHAMACOS population (Chevrier et al. 2010) were similar to those of the general U.S. population, and observed associations may be stronger in populations with higher exposure. Although, additional studies are needed to confirm our findings and to evaluate the relationships between maternal subclinical hyperthyroidism and maternal and fetal health, we believe that our results merit consideration by policy makers as well as the bromine industry.
